# Does Sex or Frailty Modify the Relationship Between Antidepressant Exposure and Dementia Risk: A Scoping Review

**DOI:** 10.1002/alz70860_106424

**Published:** 2025-12-23

**Authors:** Lucy Y Eum, Pilar Robinson Gonzalez, Sanja Stanojevic, Melissa K. Andrew, Shanna Claire Trenaman

**Affiliations:** ^1^ Dalhousie University, Halifax, NS, Canada; ^2^ Nova Scotia Health, Halifax, NS, Canada; ^3^ Canadian Consortium on Neurodegeneration in Aging, Halifax, NS, Canada; ^4^ Geriatric Medicine Research Unit, Halifax, NS, Canada

## Abstract

**Background:**

Depression is a modifiable risk factor for dementia. Prior studies on antidepressant use and dementia yielded conflicting results, potentially due to unmeasured potential confounding or potential effect modification by sex or frailty. This scoping review aimed to determine whether existing literature on antidepressant‐dementia relationship explored sex or frailty as confounders or modifiers in the antidepressant‐dementia relationship in adults over 55.

**Methods:**

We searched PubMed, MEDLINE, Embase, CINAHL, and Cochrane Library databases. Eligibility criteria were revised during screening due to the lack of eligible studies and to reduce ambiguity. Studies were included if sex or frailty of participants was described.

**Results:**

After screening 7803 titles/abstracts and 47 full texts, we included 21 observational studies. None explicitly defined or investigated sex or frailty as primary objectives. Frailty was not described or measured in any of the included studies. As for exploring sex as an effect modifier, two studies (9%) included sex as an interaction term, but one included interaction term between gender and time, rather than gender and antidepressant use. Another assessed interaction between different sexes with regards to anticholinergic‐dementia relationship but not antidepressant‐dementia relationship. As for exploring sex as a confounder, twelve studies (57%) adjusted for sex as a covariate, and the studies differed widely with regards to study characteristics, participant characteristics, and methods for analysis, including effect measures. Sex/gender binary framework predominated.

**Conclusion:**

Frailty was not explored as an effect modifier or confounder in the relationship between antidepressant use and dementia. Sex was also not explored as an effect modifier in the antidepressant‐dementia relationship. Two studies (9%) included sex as an interaction term, but they were included in such a way that the findings did not answer our research question. As for exploring sex as a confounder in the antidepressant‐dementia relationship, inconsistencies in study characteristics, participant characteristics, and methodologies (including effect measures) hinder a generalized conclusion as to whether or not sex is a confounder in the antidepressant‐dementia relationship. Future research could incorporate validated frailty measures, explore sex as an effect modifier, and include sex and gender definitions beyond the binary.

